# Interleukin-34: an important modifier in the pathogenesis of influenza pneumonia

**DOI:** 10.1186/s13054-021-03708-0

**Published:** 2021-08-04

**Authors:** Banglao Xu, Xue Lin, Yi Gong, Xiaofei Lai, Lei Ren, Ju Cao

**Affiliations:** 1 Department of Laboratory Medicine, Guangzhou First People’s Hospital, School of Medicine, South China University of Technology, Guangzhou, Guangdong China; 2 Department of Laboratory Medicine, The First Affiliated Hospital of Chongqing Medical University, No.1 Friendship Road, Yuzhong District, Chongqing, 400016 China; 3 Department of Blood Transfusion, The First Affiliated Hospital of Chongqing Medical University, No.1 Friendship Road, Yuzhong District, Chongqing, 400016 China; 4 Medical Examination Center, The First Affiliated Hospital of Chongqing Medical University, Chongqing, China

Influenza is an acute respiratory virus infection of worldwide health importance [1, 2]. Interleukin-34 (IL-34) is an important inflammatory cytokine [3, 4]. We tested here whether IL-34 contributes to the immunopathology of influenza virus infection.

Twenty-two H1N1-infected patients were enrolled, and seven H1N1 patients were diagnosed with severe pneumonia. There was a dramatic increase in serum IL-34 levels in H1N1 patients at initial diagnosis (Fig. 1a). Influenza patients with severe disease displayed significantly higher serum IL-34 levels compared with those with mild disease (Fig. 1b). Besides, serum IL-34 concentrations were significantly decreased after these patients had recovered from acute infection (Fig. 1c). Furthermore, female C57BL/6Je mice (8–10 weeks of age) were intranasally infected with 20 TCID50 of influenza virus strain A/PR/8/34 (H1N1), and we found that IL-34 levels were significantly increased in the lung and blood after H1N1 infection (Fig. 1d).

**Fig. 1:**
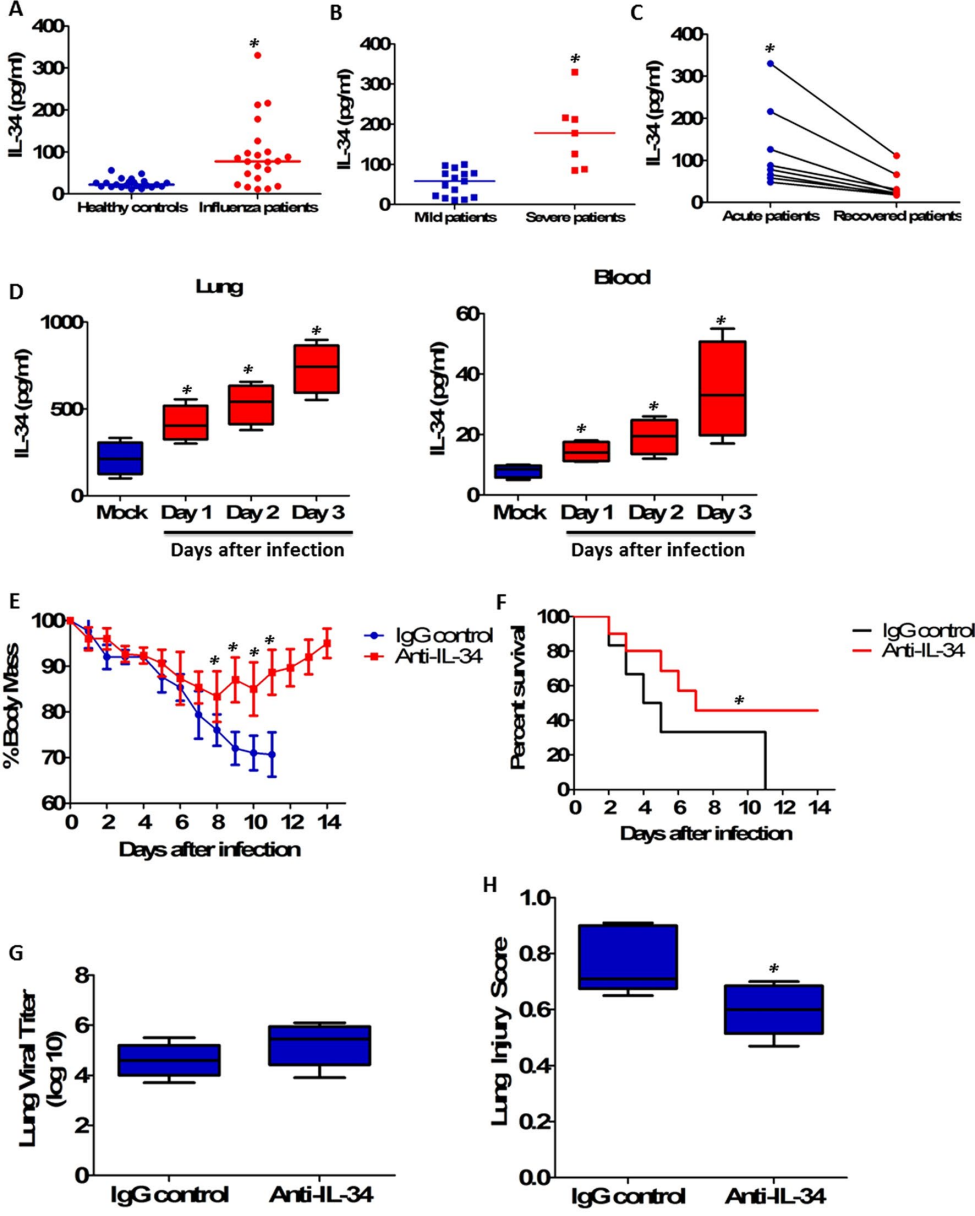

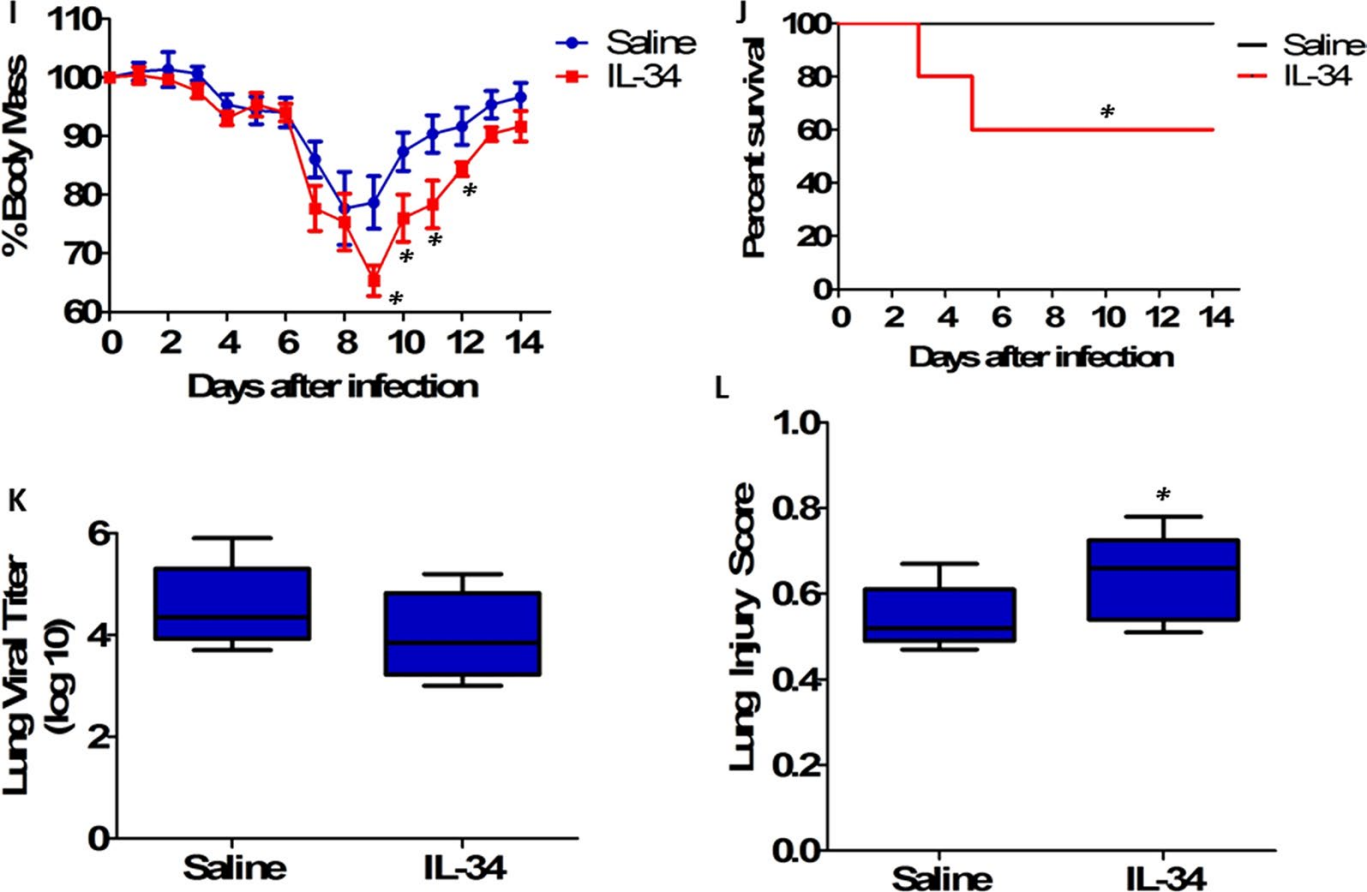
Increased IL-34 production contributed to morbidity and mortality after influenza virus infection. **a** IL-34 concentrations were assessed by ELISA in serum samples collected from 22 H1N1-infected patients upon hospital admission and from 22 healthy age- and gender-matched healthy controls. Horizontal bars represent median values, and dots represent individual participants. *P* < 0.05 when compared to healthy controls (Mann–Whitney *U* test). **b** Serum IL-34 levels in severe cases of influenza patients (*n* = 7) and mild cases of influenza patients (*n* = 15). *P* < 0.05 when compared to mild patients (Mann–Whitney *U* test). **c** Serum concentrations of IL-34 in influenza patients (*n* = 8) in the acute and recovery phases. *P* < 0.05 when compared to recovered patients (Wilcoxon signed-rank test). **d** C57BL/6Je mice (*n* = 5 per group) were infected intranasally with influenza virus PR8 (20 TCID50), and lungs and blood were collected on day 1, 2, or 3 after viral infection. Samples were assayed for IL-34 concentrations by ELISA. *P* < 0.05 when compared to mock controls (Kruskal–Wallis test followed by Dunn's multiple comparisons post test). **e** C57BL/6Je mice were intranasally infected with a lethal dose of influenza PR8 (90 TCID50). At 2 h post infection, mice were injected with 10 μg of sheep anti-mouse IL-34 antibody, followed by booster doses of 5 μg on day 2 and 4. Sheep IgG was used as a control. Weight changes of mice (*n* = 20 per group) after lethal influenza infection were calculated. *P* < 0.05 when compared to mice treated with IgG control (Mann–Whitney* U* test). **f** Survival rate was assessed daily following lethal influenza virus infection. Each group consisted of 20 mice treated with or without IL-34 blockade. Kaplan–Meier survival curves were shown and significance was determined using the log-rank test. *P* < 0.05 when compared to mice treated with IgG control (log-rank survival test). **g** In parallel, cohorts of infected mice (*n* = 5 per group) were sacrificed at day 5 after lethal influenza virus infection for measurement of virus titers in the lung, and no statistical significance was observed using Mann–Whitney *U* test. **h** The lung injury scores of lung sections from infected mice at day 5 after lethal influenza virus infection were evaluated (*n* = 5 per group). Lung injury scoring system parameters include the presence of neutrophils in the alveolar space, neutrophils in the interstitial space, hyaline membranes, proteinaceous debris filling the airspaces and alveolar septal thickening. At least 20 random regions were scored 0–2 independently and the final lung injury score was calculated. *P* < 0.05 when compared to mice treated with IgG control (Mann–Whitney* U* test). **i** C57BL/6Je mice were intranasally infected with a non-lethal dose of influenza PR8 (20 TCID50). Immediately after viral infection, mice were injected with 2 μg of recombinant mouse IL-34 protein through tail vein injection. PBS was used as saline control. Weight changes of mice (*n* = 20 per group) after non-lethal influenza infection were calculated. *P* < 0.05 when compared to mice treated with saline control (Mann–Whitney* U* test). **j** Survival rate was assessed daily following non-lethal influenza virus infection. Each group consisted of 20 mice treated with or without recombinant IL-34 protein. Kaplan–Meier survival curves were shown and significance was determined using the log-rank test. *P* < 0.05 when compared to mice treated with saline control (log-rank survival test). **k** In parallel, cohorts of infected mice (*n* = 5 per group) were sacrificed at day 5 after non-lethal influenza virus infection for measurement of virus titers in the lung, and no statistical significance was observed using Mann–Whitney *U* test. **l** The lung injury scores of lung sections from infected mice at day 5 after non-lethal influenza virus infection were evaluated (*n* = 5 per group). *P* < 0.05 when compared to mice treated with saline control (Mann–Whitney* U* test)

Next, a lethal murine model was established by intranasally infecting female C57BL/6Je mice with 90 tissue culture infectious dose 50 (TCID50) of influenza virus strain A/PR/8/34, and body weight and survival change was assessed out to 14 day. IL-34 blockade was performed by tail vein injection with 10 μg of sheep anti-mouse IL-34 antibody (R&D systems, AF5195) on day of influenza infection, followed by booster doses of 5 μg on day 2 and 4. We found that mice treated with anti-IL-34 antibodies suffered significantly less weight loss than mice treated with IgG control and started to regain body weight by day 8 after viral infection, while mice treated with IgG control continued to lose body weight until death (Fig. 1e). Furthermore, all of mice treated with IgG control were dead within 11 days post infection, whereas 50% of mice treated with anti-IL-34 antibodies survived beyond day 14 post infection (Fig. 1f). Virus titers were also measured from lung tissues 5 days post infection. Interestingly, viral titers were similar between IgG control and anti-IL-34-treated mice (Fig. 1g), suggesting that IL-34 up-regulation did not affect influenza virus replication in the lung. We assessed the histological changes of lung injury using a standardized lung injury scoring system [5], and the lung injury score was significantly lower in mice treated with anti-IL-34 antibodies as compared to mice treated with IgG control (Fig. 1h).

We further investigated the direct influence of IL-34 on non-lethal influenza virus infection, C57BL/6Je mice were intranasally infected with influenza virus strain PR8 at a dose of 20 TCID50 and then 2 μg of recombinant mouse IL-34 protein (R&D systems, 5195-ML) was inoculated into mice through tail vein injection. In mice treated with recombinant IL-34 protein, we observed a more severe decrease in weight following infection and a delayed recovery compared to mice treated with saline control (Fig. 1i). Moreover, all of mice treated with saline control survived beyond day 14 post non-lethal influenza virus infection, whereas 40% mice treated with recombinant IL-34 protein were dead within 5 days post infection (Fig. 1j). We next examined viral clearance by quantitating lung viral titers in mice treated with or without IL-34 on day 5 after non-lethal influenza virus infection, and there was no significant difference in viral titers (Fig. 1k). Histological evaluation revealed that the lung injury score was significantly higher in IL-34-treated mice as compared to saline-treated mice after non-lethal influenza virus infection (Fig. 1l).

Collectively, our data demonstrated a detrimental role of IL-34 in the immunopathology of influenza virus infection. We therefore speculate that excessive IL-34 amounts may have important implications in the development of influenza virus-induced lung injury.

## Data Availability

Data sharing is offered under the format of collaborative projects. Proposals can be directed to the corresponding author.
